# Clinical Classification and Collateral Circulation in Chronic Cerebrospinal Venous Insufficiency

**DOI:** 10.3389/fneur.2020.00913

**Published:** 2020-09-23

**Authors:** Zhongao Wang, Jiayue Ding, Chaobo Bai, Yuchuan Ding, Xunming Ji, Ran Meng

**Affiliations:** ^1^Department of Neurology, Xuanwu Hospital, Capital Medical University, Beijing, China; ^2^Advanced Center of Stroke, Beijing Institute for Brain Disorders, Beijing, China; ^3^Department of China-America Institute of Neuroscience, Xuanwu Hospital, Capital Medical University, Beijing, China; ^4^Department of Neurosurgery, Wayne State University School of Medicine, Detroit, MI, United States; ^5^Department of Neurosurgery, Xuanwu Hospital, Capital Medical University, Beijing, China

**Keywords:** chronic cerebrospinal venous insufficiency, internal jugular vein, clinical classification, vertebral venous system, collateral circulation

## Abstract

**Background:** As an indispensable part of the cerebral venous system, the extracranial cerebrospinal venous system is not fully recognized. This study aimed to analyze the clinical classification and imaging characteristics of chronic cerebrospinal venous insufficiency (CCSVI) quantitatively.

**Methods:** A total of 128 patients, who were diagnosed as CCSVI by jugular ultrasound and contrast-enhanced magnetic resonance venography (CE-MRV), were enrolled from May 2018 through May 2019. For the patients with possible extraluminal compression, computed tomography venography (CTV) was applied to estimate the degree of internal jugular venous stenosis (IJVS) and rank the vertebral venous collateral circulation.

**Results:** The causes of extraluminal compression induced IJVS included osseous compression (78.95%), carotid artery (24.21%), sternocleidomastoid muscle (5.79%), swollen lymph node (1.05%), and unknown reasons (5.26%). The subtypes of non-compression CCSVI included the high jugular bulb (77.27%), fenestration of the internal jugular vein (IJV) (7.27%), internal jugular phlebectasia (2.73%), tortuous IJV (0.91%), IJV thrombosis (14.55%), and elongated venous valves with/without erythrocyte aggregation (13.64%). For extraluminal compression induced IJVS, the ratio of severe vertebral venous expansion was higher in the severe IJVS group than that in the mild IJVS group (*p* < 0.001). The IJVS degree was higher in the severe vertebral venous expansion group than in the mild vertebral venous expansion group (*p* < 0.001).

**Conclusions:** A multimodal diagnostic system is necessary to improve the diagnostic accuracy of CCSVI. The vertebral venous system is an important collateral circulation for CCSVI, which may be a promising indicator for evaluating IJVS degree.

## Introduction

The cerebrospinal venous system is a crucial channel for the cerebral venous outflow, which plays an important role in transporting metabolic wastes, collecting cerebral spinal fluid, and regulating intracranial pressure. Intracranial venous outflow insufficiency has attracted much attention in clinical practice due to the typical symptoms. However, as an indispensable part of the cerebral venous system, the disturbance of the extracranial cerebrospinal venous system is far from fully recognized by the non-specific clinical presentations and inadequate awareness (1, 2).

Previous studies revealed that CCSVI might be related to Alzheimer's disease, Parkinson's disease, multiple sclerosis, migraine, and Ménière disease with the IJVs and/or azygous vein outflow disturbance (3–7). Our previous study showed that CCSVI might be relevant to an independent disease entity, with non-focal neurological symptoms such as sleep disturbance, tinnitus, head noise, dizziness, and headache whereas without specific clinical signs and imaging findings in the brain, misdiagnosis or missed diagnosis is common. The lesions for the type of CCSVI are commonly present in IJV with abnormally dilated paraspinal collateral veins. The diagnostic delay could interfere with the normal life of the involved patients for a long time (8). Moreover, the clinical symptoms could be relieved after the correction of CCSVI with medical and surgical treatment (9, 10).

However, it is still controversial whether CCSVI is normal variation or pathological change, because of the superficial position of IJV easily being invaded, the debatable significance of cerebrospinal venous collateral circulation, and the indefinite relationship between CCSVI and clinical symptoms (11, 12). It is difficult to carry out in-depth studies due to the lack of a systematic clinical classification and a universal diagnostic criterion based on imaging evidence. Previous studies have explored the hemodynamic change and the collateral circulation in patients with CCSVI by ultrasound and magnetic resonance imaging (MRI) (13–18). However, no report included the relationship between IJVS degree and vertebral venous collateral circulation. In this study, we aimed to describe the clinical classification of CCSVI systematically with a multimodal diagnostic system, and further estimate the correlation between IJVS and collateral circulation quantitatively with a CTV scan.

## Materials and Methods

From May 2018 to May 2019, a total of 128 patients, who were diagnosed with CCSVI in the neurology department of Xuanwu Hospital, Capital Medical University, were enrolled in this retrospective study, which was approved by the Institutional Ethic Committee of Xuanwu Hospital, Capital Medical University. All the participants gave written informed consent before any study-specific procedure in accordance with the Declaration of Helsinki.

### Population

Patients were enrolled in this study according to the following inclusion criteria: (a) age from 18 to 80 years; (b) internal jugular anomalies confirmed by CE-MRV/CTV/jugular ultrasound; (c) with non-focal neurological deficits; and (d) with abnormally dilated paraspinal collateral veins.

Exclusion criteria included: (a) patients with definite intracranial/extracranial arterial stenosis; (b) a history of brain infarction or cerebral hemorrhage; (c) with other cerebral venous disorders; (d) with obstruction of the superior vena cava or severe cardiac dysfunction which could interfere with cerebral venous return; (e) comorbid with austere diseases; or (f) with severe liver and renal dysfunction or malignant tumor.

### Diagnosis

**Figure 1:**
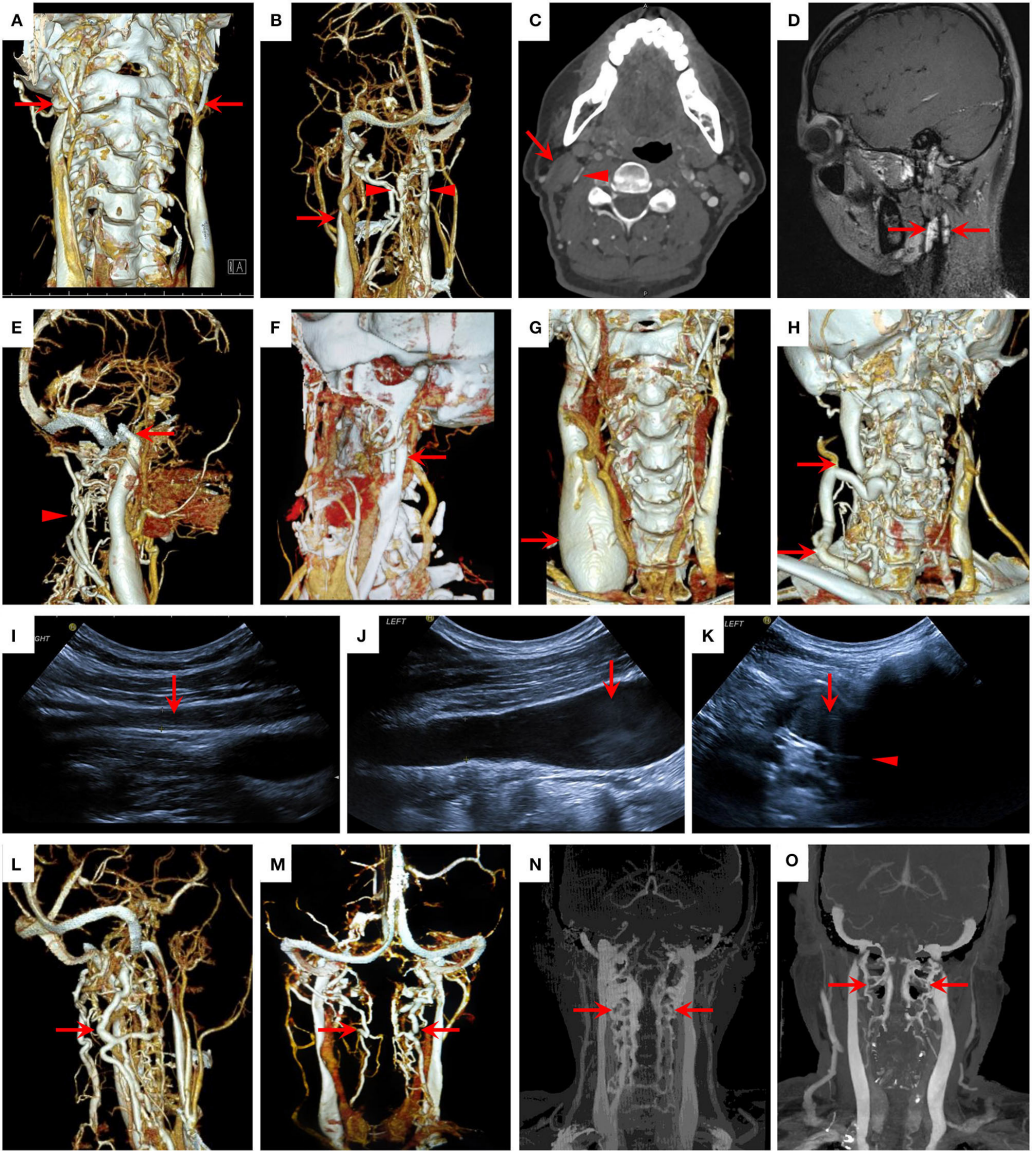
Clinical classification and collateral circulation of CCSVI. **(A–D)** CCSVI by extra-luminal compression in 3D-computed tomography venography (3D-CTV) and contrast-enhanced magnetic resonance imaging (CE-MRI) maps. **(A)** Bilateral IJVS compressed by the transverse process of C1 and styloid process at the J3 segments (red arrow); **(B)** Left IJVS compressed by carotid artery (red arrow) at the J2 segment with abnormally dilated paraspinal collateral veins (red triangle); **(C)** Right IJVS (red triangle) compressed by sternocleidomastoid muscle (red arrow) at the J2 segment; **(D)** IJVS compressed by swollen lymph nodes (red arrow) at the J2 segment in CE-MRI. **(E–H)** CCSVI by malformation in 3D-CTV maps. **(E)** Right high jugular bulb (red arrow) at the J3 segment with abnormally dilated paraspinal collateral veins (red triangle); **(F)** Fenestration of left IJV at the J2 segment (red arrow); **(G)** Right internal jugular phlebectasia at the J1 segment (red arrow); **(H)** Right tortuous IJV from the J3 to J1 segments (red arrow). **(I–K)** CCSVI by intraluminal anomaly in jugular ultrasound maps. **(I)** IJV thrombosis from the J3 to J1 segments (red arrow); **(J–K)** Elongated venous valve (red triangle) in IJV with erythrocyte aggregation (red arrow) at the J1 segment. **(L–O)** Degree of vertebral venous expansion in 3D-CTV maps. **(L)** Severe external vertebral venous expansion (red arrow); **(M)** Mild external vertebral venous expansion (red arrow); **(N)** Severe internal vertebral venous expansion (red arrow); **(O)** Mild internal vertebral venous expansion (red arrow).

The diagnosis criteria of extraluminal compression induced IJVS included: (a) the residual cross-sectional area <50% of the estimated cross-sectional area at stenosis segment; and (b) with abnormally dilated paraspinal collateral veins (19, 20) (Figures 1A–D).

The diagnosis for non-compression CCSVI included: (a) the intraluminal anomaly of the IJV was diagnosed by jugular ultrasound and CE-MRV; (b) the malformation related CCSVI was diagnosed by jugular ultrasound, CE-MRV, and CTV; (c) the high jugular bulb was confirmed by the thin layer basicranial scanning of computerized tomography (CT); and (d) with abnormally dilated paraspinal collateral veins (Figures 1E–K).

### Assessment

To investigate the correlation of IJVS to the expansion degree and patterns of vertebral venous collateral circulation, a total of 92 patients with IJVS who underwent a CTV scan were selected. The IJVS degree and the expansion degree and patterns of vertebral venous collateral circulation on CTV maps were evaluated by two imaging experts who did not know the clinical data of the patients. Differences between the two experts were resolved by discussion, or, another expert would decide the result if the consensus could not be reached.

Calculation formulas for IJVS were as follows: Unilateral IJVS degree: A_1_ = [1–(S_1_/S_2_)]×100%; S_2_ = (S_3_+S_4_)/2. (S_1_: residual cross-sectional area at stenosis segment, S_2_: estimated cross-sectional area at stenosis segment, S_3_: normal cross-sectional area at upper slice of stenosis segment, S_4_: normal cross-sectional area at lower slice of stenosis segment). A_1_ ranging from 50 to 74.99% was defined as mild IJVS and 75 to 100% was severe IJVS. For the IJV with multi-segments stenosis, S_1_ was measured at the narrowest slice.

To study the influence of bilateral IJVS, the weighted bilateral IJVS degree was defined when considering the unilateral dominance of IJV. The weighted bilateral IJVS degree (A_2_) was the sum of bilateral A_3_ (or ${\text{A}}_{3}^{\prime }$). For the patients with bilateral IJVS, weighted unilateral IJVS degree: A_3_ = A_1_×S_2_/(S_2_ + ${\text{S}}_{2}^{\prime }$) × 100%. For the patients with unilateral IJVS, weighted unilateral IJVS degree: ${\text{A}}_{3}^{\prime }=$A_1_×S_2_/(S_2_ + S_c_) × 100%. (${\text{S}}_{2}^{\prime }$: estimated cross-sectional area at contralateral stenosis segment, S_c_ = normal cross-sectional area at contralateral segment).

In this study, the opening patterns of vertebral venous collateral circulation can be divided into two levels, collaborative internal-external vertebral veins and isolated internal or external vertebral veins. Internal vertebral veins were defined as the paraspinal collateral veins running through the spinal canal and the transverse process of the cervical area shown in CTV, including the anterior internal vertebral plexus and the posterior internal vertebral plexus. External vertebral veins were defined as the paraspinal collateral veins posterior to the spinal canal depicted by CTV, namely the suboccipital venous plexus (21, 22).

Moreover, the degree of vertebral venous expansion was divided into two levels, severe and mild. The former included at least one side of the vertebral venous collateral circulations having severe expansion and the latter was both sides of the vertebral venous collateral circulations having mild expansion. The definitions for the expansion degree of external and internal vertebral veins were as follows: severe external vertebral venous expansion was the maximal cross-sectional area of the external vertebral vein ≥25% of the estimated cross-sectional area at the stenosis segment or the normal cross-sectional area at J3 of the adjacent IJV, and mild external vertebral venous expansion was <25%. Severe internal vertebral venous expansion was the length of the internal vertebral vein ≥50% of the length of the adjacent IJV with mass expansion at the atlantoaxial vertebra, and mild internal vertebral venous expansion was <50% (19, 20) (Figures 1L–O).

### Treatment Strategy

Standard and individualized therapy was administered according to the etiology of each patient, including anticoagulant/ antiplatelet treatment, oxygen inhalation, dehydration, and other symptomatic therapy to relieve non-specific symptoms. Given lack of a universal indication and consensus on treatment strategy, the interventional and surgical therapies were applied only when a significant obstruction of IJV was confirmed and the patient strongly requested an active surgical intervention, after adequate preoperative preparation (9, 20).

### Statistical Analysis

SPSS Version 19.0 was used for statistical analyses in this study (SPSS, Inc., Chicago, IL). Continuous variables were expressed as mean ± standard deviation (SD) when following a Gaussian distribution and analyzed by *t*-test; otherwise, they were presented as median (IQR) and analyzed by the Mann–Whitney *U*-test. Categorical variables were depicted as number (percentage) and analyzed by the Chi-square or Fisher exact test. A two-tailed *P* < 0.05 was considered as statistical significance.

## Results

### Demographics

A total of 128 patients (60 males and 68 females) diagnosed with CCSVI were enrolled in this retrospective study consecutively. The average age was 55.63 ± 12.34 years (range from 19 to 79). The clinical symptoms included sleep disturbance, head noise, tinnitus, dizziness, and headache. No positive neurological sign was detected. Details in Table 1.

**Table 1:** Clinical features of CCSVI.

**Clinical features**	
**Demographics**	
No. of patients	128
Gender (male/female)	60/68
Age (years) (Mean ± SD)	55.63 ± 12.34
Symptoms onset age (years) (Mean ± SD)	48.06 ± 14.22
Onset-to-door time (months) (Median, IQR)	36 (5.75–120)
**Clinical symptoms (No., %)** ***n*** **=** **128**	
Sleep disturbance	88 (68.75)
Head noise	71 (55.47)
Tinnitus	66 (51.56)
Dizziness	63 (49.22)
Headache	54 (42.19)
Hearing disorder	45 (35.16)
Visual disorder	42 (32.81)
Dry or puffy eyes	42 (32.81)
Neck discomfort	41 (32.03)
Nausea or vomiting	28 (21.88)
Anxiety or depression	23 (17.97)
Vertigo	17 (13.28)
Subjective memory decline	11 (8.59)
Number of manifestations (Median, IQR)	5 (3–6)

*CCSVI, chronic cerebrospinal venous insufficiency; IQR, inter quartile range; SD, standard deviation*.

### Imaging Presentations

Of the 128 patients, 100 cases (78.13%) had bilateral CCSVI and the remaining were unilateral (12 right, 16 left). In total, 228 out of the 256 sides of IJVs (89.06%) presented with CCSVI.

#### Involved Segments of CCSVI

The involved segments of CCSVI included J1, J2, and J3 segments. J3 was the most involved segment (88.16%), followed by J2 (25.88%) and J1 (10.09%). Interestingly, some CCSVI presented multi-segment lesions in one IJV. Of the 228 sides of involved IJVs, 45 (19.74%) were two-segment lesions and 5 (2.19%) were three-segment lesions.

#### Clinical Classification of CCSVI

The clinical classification of CCSVI can be divided into extraluminal compression (83.33%) and non-compression (48.25%) subsets. The osseous compression accounted for the predominant proportion in the extraluminal compression subset (78.95%), in which 81.33% was compressed by the transverse process of C1 with the styloid process and 18.67% was compressed by the isolated transverse process of C1. Other causes of extraluminal compression included carotid artery (24.21%), sternocleidomastoid muscle (5.79%), swollen lymph node (1.05%), and unknown reasons (5.26%) (Figures 1A–D). As for the reasons of non-compression CCSVI, malformation related CCSVI included high jugular bulb (77.27%), fenestration of IJV (7.27%), internal jugular phlebectasia (2.73%), and tortuous IJV (0.91%) (Figures 1E–H). Furthermore, intraluminal anomaly related CCSVI included IJV thrombosis (14.55%) and elongated venous valves with/without erythrocyte aggregation (13.64%) (Figures 1I–K). Susceptible segments were shown in Table 2.

**Table 2:** Clinical classification and segments of CCSVI.

	**J3**	**J2**	**J1**
**Extraluminal compressive IJV (sides, %)** ***n*** **=** **190**			
Osseous compression	150 (78.95)	0 (0.00)	0 (0.00)
Carotid artery	0 (0.00)	46 (24.21)	0 (0.00)
Sternocleidomastoid muscle	1 (0.53)	8 (4.21)	2 (1.05)
Swollen lymph node	0 (0.00)	2 (1.05)	0 (0.00)
Unknown reason	8 (4.21)	1 (0.53)	1 (0.53)
**Non-compressive IJV (sides, %)** ***n*** **=** **110**			
High jugular bulb	85 (77.27)	0 (0.00)	0 (0.00)
Fenestration	7 (6.36)	1 (0.91)	0 (0.00)
Internal jugular phlebectasia	0 (0.00)	0 (0.00)	3 (2.73)
Tortuous IJV^§^	1 (0.91)	1 (0.91)	1 (0.91)
IJV thrombosis^†^	16 (14.55)	2 (1.82)	2 (1.82)
Elongated venous valves with/without erythrocyte aggregation	0 (0.00)	0 (0.00)	15 (13.64)

*CCSVI, chronic cerebrospinal venous insufficiency; IJV, internal jugular vein*.

§ *Tortuous IJV from the J3 to J1 segments was present in 1 side of IJV*.

† *IJV thrombosis from the J3 to J1 segments was present in 2 sides of IJVs*.

#### Features of Vertebral Venous Collateral Circulation

Of the 128 patients, 46 (35.94%) had external vertebral veins alone, 21 (16.41%) had internal vertebral veins alone and 61 (47.66%) had collaborative internal-external vertebral veins. As for the degree of vertebral venous expansion, 58 (45.31%) had severe vertebral venous expansion and the remaining 70 (54.69%) had mild vertebral venous expansion.

#### Results of the Jugular Ultrasound

The jugular ultrasound results (supine position) of the IJVs in the extraluminal compression subset were as follows: the mean diameter was 4.48 ± 2.24 mm at the stenosis segment and 7.31 ± 2.05 mm at the non-stenosis segment (*p* < 0.001). The flow velocity was 99.17 ± 43.20 cm/s at the stenosis segment and 39.11 ± 22.01 cm/s at the non-stenosis segment (*p* < 0.001).

### The Vertebral Venous Collateral Circulation for CCSVI

#### Correlation of IJVS to the Degree of Vertebral Venous Expansion

As is shown in Table 3, the correlations of IJVS to the degree of vertebral venous expansion among the 92 patients who underwent a CTV scan were as follows: the unilateral IJVS degree and the weighted bilateral IJVS degree were higher in the severe vertebral venous expansion group than in the mild vertebral venous expansion group (*p* < 0.001). Of the 62 sides of IJVs with severe stenosis, 50 (80.65%) had severe vertebral venous expansion. Of the 112 sides of IJVs with mild stenosis, 42 (37.50%) had severe vertebral venous expansion (*p* < 0.001).

**Table 3:** Correlation of IJVS to the degree of vertebral venous expansion.

	**Severe vertebral venous expansion**	**Mild vertebral venous expansion**	***p*-value**
Unilateral IJVS degree (%) (Mean ± SD)	72.82 ± 12.80	60.90 ± 11.88	<0.001
Weighted bilateral IJVS degree (%) (Mean ± SD)	69.56 ± 16.55	48.69 ± 19.00	<0.001
**Extraluminal compressive IJV (sides, %)**			
Severe stenosis	50 (80.65)	12 (19.35)	<0.001
Mild stenosis	42 (37.50)	70 (62.50)	
**Bilateral IJVS patient (No., %)**			
Bilateral severe IJVS	15 (83.33)	3 (16.67)	<0.001
One-side severe IJVS	19 (86.36)	3 (13.64)	
Bilateral mild IJVS	8 (26.67)	22 (73.33)	

*IJVS, internal jugular venous stenosis; IJV, internal jugular vein; SD, standard deviation*.

Furthermore, of the 92 patients, 70 with bilateral IJVS were divided according to both the side and degree of IJVS to investigate their influence on the degree of vertebral venous expansion. Details in Table 3. The ratios of patients with severe vertebral venous expansion in both the bilateral severe IJVS group (83.33%) and the one-side severe IJVS group (86.36%) were more than that in the bilateral mild IJVS group (26.67%) (*p* < 0.001). However, no statistical difference was noticed between the bilateral severe IJVS group (83.33%) and the one-side severe IJVS group (86.36%) (*p* > 0.05).

#### Correlation of IJVS to the Pattern of Vertebral Venous Collateral Circulation

As is shown in Table 4, the correlations of IJVS to the pattern of vertebral venous collateral circulation among the 92 patients who underwent a CTV scan were as follows: the unilateral IJVS degree (*p* = 0.01) and the weighted bilateral IJVS degree (*p* = 0.02) were higher in the collaborative internal-external vertebral veins group than in the isolated internal or external vertebral vein group. Of the 62 sides of IJVs with severe stenosis, 40 (64.52%) had collaborative internal-external vertebral veins. Of the 112 sides of IJVs with mild stenosis, 46 (41.07%) had collaborative internal-external vertebral veins (*p* = 0.003).

**Table 4:** Correlation of IJVS to the pattern of vertebral venous collateral circulation.

	**Collaborative internal-external vertebral veins**	**Internal or external vertebral vein**	***p*-value**
Unilateral IJVS degree (%) (Mean ± SD)	69.90 ± 13.90	64.57 ± 13.06	0.010
Weighted bilateral IJVS degree (%) (Mean ± SD)	64.50 ± 20.13	54.64 ± 20.03	0.021
**Extraluminal compressive IJV (sides, %)**			
Severe stenosis	40 (64.52)	22 (35.48)	0.003
Mild stenosis	46 (41.07)	66 (58.93)	
**Bilateral IJVS patient (No., %)**			
Bilateral severe IJVS	11 (61.11)	7 (38.89)	0.006
One-side severe IJVS	16 (72.73)	6 (27.27)	
Bilateral mild IJVS	9 (30.00)	21 (70.00)	

*IJVS, internal jugular venous stenosis; IJV, internal jugular vein; SD, standard deviation*.

Similarly, the 70 patients with bilateral IJVS were also divided according to both the side and degree of IJVS to further investigate their influence on the pattern of vertebral venous collateral circulation. Details in Table 4. The ratio of patients with collaborative internal-external vertebral veins in the one-side severe IJVS group (72.73%) was more than in the bilateral mild IJVS group (30.00%) (*p* = 0.002). However, there was no statistical difference between the bilateral severe IJVS group (61.11%) and the bilateral mild IJVS group (30.00%) (*p* = 0.03) or between the bilateral severe IJVS group (61.11%) and the one-side severe IJVS group (72.73%) (p > 0.05).

### Treatment Outcome

**Figure 2:**
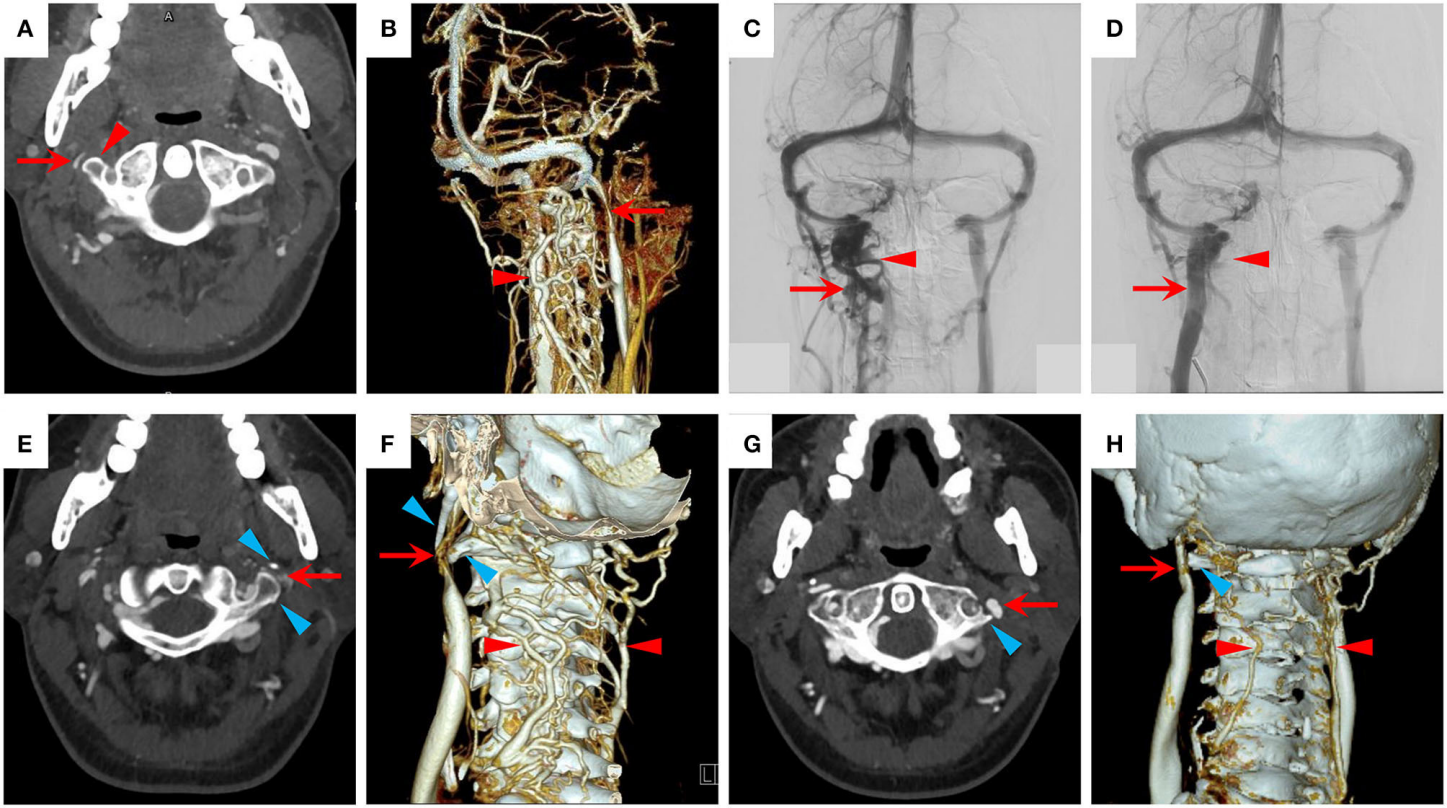
Treatment outcome of patients with IJVS. **(A–D)** The treatment outcome of a patient with right IJVS. **(A)** A severe stenosis at right IJV (red arrow) by the transverse process of C1 (red triangle) in CTV map; **(B,C)** A severe stenosis at right IJV (red arrow) with severe vertebral venous expansion (red triangle) shown in 3D-CTV and digital subtraction angiography (DSA) maps; **(D)** The right IJVS was corrected after stenting therapy (red arrow) and the abnormally dilated paraspinal collateral veins disappeared (red triangle) in the DSA map. **(E–H)** The treatment outcome of a patient with left IJVS. **(E,F)** A severe IJVS at left IJV (red arrow) by the compression of transverse process of C1 and styloid process (blue triangle) with abnormally dilated external vertebral veins (red triangle) in CTV maps; **(G,H)** The IJVS was corrected (red arrow) and the compensatory external vertebral veins attenuated (red triangle) after surgical removal of the transverse process of C1 and styloid process (blue triangle) in postoperative CTV maps.

The symptoms of patients with non-compression CCSVI could be relieved after medical therapy. As for the extraluminal compression induced IJVS, two patients underwent surgical treatment and got significant relief for their clinical symptoms. A 28-year-old male, who had complained of dizziness, visual disorder, puffy eyes, and sleep disturbance for 5 years, was diagnosed with IJVS by CTV, which showed a severe stenosis at the right IJV by the transverse process of C1 with severe vertebral venous expansion. The IJVS was corrected after a stent was implanted at the stenotic segment and the abnormally dilated paraspinal collateral veins disappeared immediately (Figures 2A–D). Moreover, the dizziness, puffy eyes, and sleep disturbance gradually attenuated at the 3-month follow-up. Additionally, a 49-year-old male had complained of head noise and dizziness for 3 years. A severe IJVS at the left IJV was detected by CTV, being attributed to the transverse process of C1 and styloid process, with abnormally dilated external vertebral veins. After the surgical removal of the transverse process of C1 and styloid process and the conduction of balloon angioplasty, postoperative CTV showed that the IJVS was corrected and the compensatory external vertebral veins visibly attenuated (Figures 2E–H). The head noise and dizziness had disappeared completely at the 1-year follow-up.

## Discussion

### Anastomoses Between Intracranial and Extracranial Veins

IJV plays an important role for cerebral venous outflow. Previous studies revealed that IJV and other paraspinal collateral veins formed a compensatory extracranial cerebrospinal venous system to guarantee an adequate cerebral venous return (21, 22). These anastomoses, connecting intracranial and extracranial veins, can be divided into four groups according to their location at the vertebral column, including the suboccipital venous plexus, the posterior internal vertebral plexus, the anterior internal vertebral plexus, and the anterior external vertebral plexus (21, 22). The collateral veins expanded significantly in patients with CCSVI compared with the healthy controls, and decreased evidently after the correcting of jugular outflow, which proved their significant compensatory capacity (13, 14).

Considering the complex anastomoses among the collateral veins, many studies used the method, calculating the extracranial cerebrospinal venous outflow normalized to cerebral arterial inflow, to evaluate the collateral circulation quantitatively (13, 15, 16). An ultrasound model was developed to assess the hemodynamic change in patients with CCSVI, which proved the critical role of the collateral vessels to drain the cerebral venous return (13). The accuracy of the ultrasound relies on the operator's experience, and the irregular internal vertebral veins and minor vessels could not be well-detected by the jugular ultrasound (23, 24). What is more, some studies used the MRI to measure the anatomical and hemodynamic changes of the cerebrospinal veins for CCSVI. The time-of-flight MRI was used to diagnose the IJVS and the phase-contrast MRI was used to quantify cerebrospinal arterio-venous flow. The results also showed an increased flow in the paraspinal collateral veins for CCSVI (17, 18). The IJVS was assessed by an absolute cross-sectional area, which might ignore the frequent individual variation of IJV. The accuracy for measuring the cervical venous flow is still unknown with only MRI being used (24). CTV, with high spatial resolution and convenience, can quantify the IJVS degree and vertebral venous expansion accurately. The digital subtraction radiography technique and the three-dimensional reconstruction image can visualize the cerebrospinal veins directly (24). Our study showed a correlation between IJVS degree and vertebral venous expansion, but failed to quantify the anterior external vertebral plexus, draining the cavernous sinuses, condylar veins, and thyroid veins, which might affect the assessment of vertebral venous collateral circulation. Recently, a new model was developed which took the advantages of both the MRI and ultrasound comprehensively, and revealed a high degree of consistency between the clinical examination results and the simulated data for CCSVI (23). Hopefully, a multimodal imaging technique may propel further research on the pathophysiologic mechanism of CCSVI with the combination of ultrasound, MRI, and CTV (25).

### The Adverse Impact of CCSVI

It is well-acknowledged that pressing on one side of IJVs can cause a rapid increase of intracranial pressure during lumbar puncture. A study reported that the severity of migraine increased dramatically after compressing over bilateral IJVs for 30 sec, which proved the adverse impacts of CCSVI (5). Studies showed that the dominant drainage was IJV in supine position and was paraspinal collateral vein in upright position in the normal population (26–28). Therefore, CCSVI may not be harmful to the patients in the upright position such as daytime routine activities. However, the cerebral blood outflow through IJV, which is supposed to be the main drainage route, will be blocked by the lesions in the supine position, such as when sleeping. The blood flow of the paraspinal collateral veins may be not enough to completely compensate that of IJVs (27). It could be inferred that gravity assists the vertebral venous return in the upright position and the central venous pressure in the upright position is lower than that in the supine position, which can prevent venous reflux (29). Whereas, as there is no valve in the paraspinal collateral veins, the loss of gravity assisting venous return, and the increase of central venous pressure will increase the risk of cerebral venous stasis and reflux in the supine position by vertebral venous drainage (29). Additionally, it was reported that the clearance rate of metabolic waste in the brain increased significantly during sleep (30). Moreover, the damaged clearance of metabolic waste through the cerebral venous system may contribute to the pathogenesis of vascular cognitive impairment (31, 32). In this study, the decreased diameter and the increased flow velocity at the stenosis segment showed the significantly disturbed jugular venous outflow in patients with CCSVI (33). The abnormal collateral vessels, the reduced cerebral perfusion, and the chronic clinical symptoms could be relieved after the correction of CCSVI, which further indicated the significance of IJV drainage (14). Therefore, IJV with valves will keep a steady venous return when sleeping in the supine position and long-term CCSVI may result in brain damage.

### Imaging Techniques for Detecting CCSVI

The clinical classification of CCSVI is complicated. The lesions may involve each segment from J1 to J3, and some patients may have bilateral IJV lesions and combined lesions involving multiple segments at one IJV, which indicate that a part of CCSVI might be the result of multiple factors. There are many imaging techniques for the diagnosis of CCSVI, however, the sensitivity and specificity of them varied with different settings. Jugular ultrasound had the advantage of high accuracy for detecting intraluminal anomalies of IJV and assessing the blood flow dynamically. Although, the repeatability and accuracy for measuring the diameter and flow velocity will be impacted by multiple factors. The internal vertebral vein in the spinal canal is difficult to detect and estimate quantitatively by ultrasound as it is surrounded by cervical vertebrae and its shape is irregular (34). CE-MRV can estimate the location of each vein spatially and comprehensively, but it will amplify the severity of IJVS. Instead, CTV has fast scanning speed and high spatial resolution, which will show the cross-sectional area of IJV and the location of paraspinal collateral veins accurately (24). CTV is only used for assessing the IJVS degree quantitatively and ranking the paraspinal collateral veins when necessary due to its radioactivity. However, it is difficult to make a definite diagnosis by a single imaging technique because of the complicated clinical classification for CCSVI. A multimodal diagnostic system is required when necessary to improve the diagnostic accuracy (25).

### The Significance of Vertebral Venous Collateral Circulation

The pathological significance of paraspinal collateral veins is still controversial. It was reported in one study that IJVS and the vertebral venous expansion could also exist in the normal population (11). However, in this study, only CE-MRV was used to evaluate the IJVS degree and it could overestimate the severity of the stenosis (24). Moreover, the expansion degree of external vertebral veins in this study was milder than that of our patients and no obvious internal vertebral venous expansion was detected as is shown in the typical images. By contrast, the ratio of patients with internal vertebral veins was as high as 64.06%. In addition, another study reported that paraspinal collateral veins could be the major channels of cerebral venous outflow in about 6% of the normal population (28). However, the ratio of patients with severe vertebral venous expansion was up to 45.31% in our study. Whereby, there were significant differences between the patients in our study and the normal population in the aforementioned two studies. As a result, it can be concluded that the abnormally dilated paraspinal collateral veins in our study is due to pathological collateral circulation during CCSVI rather than physiological variation.

### The Correlation Between IJVS and Vertebral Venous Collateral Circulation

The correlation between IJVS and paraspinal collateral veins is not fully recognized. Our results showed that the ratios of severe vertebral venous expansion and collaborative internal-external vertebral veins were higher in the severe IJVS group than in the mild IJVS group. After the correction of IJVS, the abnormally dilated paraspinal collateral veins visibly attenuated, which was consistent with the result in the previous study (14). These results suggested that the vertebral venous system was an important collateral circulation for patients with IJVS in the supine position. Moreover, the ratios of severe vertebral venous expansion and collaborative internal-external vertebral veins were lower in the bilateral mild IJVS group when compared to either the bilateral severe IJVS group or the one-side severe IJVS group. So, one-side severe IJVS might be enough to have an obvious impact on the hemodynamics of the extracranial cerebrospinal venous system. Additionally, the results showed that the weighted bilateral IJVS degree was higher in the severe vertebral venous expansion group than in the mild vertebral venous expansion group. Also, it was higher in the collaborative internal-external vertebral veins group than in the isolated internal or external vertebral vein group as well. Consequently, the expansion degree and patterns of vertebral venous collateral circulation may be promising indirect indicators for evaluating the IJVS degree.

### Limitations

There are some limitations in this study. Firstly, this is a single-center study and the sample size is not large enough. Secondly, it is difficult to make a universal diagnostic criterion for CCSVI due to the absence of data in the normal population. Thirdly, the collateral circulation for cerebrospinal venous system is complex. We failed to quantify the anterior external vertebral plexus with CTV, draining the cavernous sinuses, condylar veins, and thyroid veins, which might affect the assessment of vertebral venous collateral circulation. The method, calculating the extracranial cerebrospinal venous outflow normalized to cerebral arterial inflow with multimodal imaging technique, could be promising for exploring the hemodynamic change of CCSVI in future studies. Moreover, the treatment strategy of CCSVI is still controversial due to the lack of a universal indication and consensus. Our study cannot prove the efficacy and safety of the surgical treatment because only two patients underwent surgical intervention in this cohort. Further in-depth research is needed to investigate the pathophysiology of CCSVI. Multicenter studies with a large sample size and a universal diagnostic criterion are required in the future.

## Conclusions

The clinical classification of CCSVI is complicated, whereby a multimodal diagnostic system is required to improve the diagnostic accuracy. The vertebral venous system is an important compensating system for CCSVI, which may be a promising indirect indicator for evaluating the severity of IJVS.

## Data Availability Statement

The raw data supporting the conclusions of this article will be made available by the authors, without undue reservation.

## Ethics Statement

The studies involving human participants were reviewed and approved by the Institutional Ethic Committee of Xuanwu Hospital, Capital Medical University. The patients/participants provided their written informed consent to participate in this study. Written informed consent was obtained from the individual(s) for the publication of any potentially identifiable images or data included in this article.

## Author Contributions

ZW and JD wrote the first draft of the manuscript. ZW and CB performed the material preparation, data collection, and statistical analysis. RM and YD wrote sections of the manuscript and contributed to manuscript revisions. XJ and RM contributed to the conception and design of the study. RM takes full responsibility for the data, the analyses and interpretation, and the conduct of the research. All authors have read and approved the submitted version.

## Conflict of Interest

The authors declare that the research was conducted in the absence of any commercial or financial relationships that could be construed as a potential conflict of interest.

## Abbreviations

CCSVI: chronic cerebrospinal venous insufficiency
CE-MRI: contrast-enhanced magnetic resonance imaging
CE-MRV: contrast-enhanced magnetic resonance venography
CT: computerized tomography
CTV: computed tomography venography
IJV: internal jugular vein
IJVS: internal jugular venous stenosis
MRI: magnetic resonance imaging.
